# On Alternative Uses of Structural Compliance for the Development of Adaptive Robot Grippers and Hands

**DOI:** 10.3389/fnbot.2019.00091

**Published:** 2019-11-07

**Authors:** Che-Ming Chang, Lucas Gerez, Nathan Elangovan, Agisilaos Zisimatos, Minas Liarokapis

**Affiliations:** ^1^New Dexterity Research Team, Department of Mechanical Engineering, University of Auckland, Auckland, New Zealand; ^2^Department of Electrical Engineering, National Technical University of Athens, Athens, Greece

**Keywords:** structural compliance, adaptive grippers, grasping, manipulation, dexterity

## Abstract

Adaptive robot hands are typically created by introducing structural compliance either in their joints (e.g., implementation of flexures joints) or in their finger-pads. In this paper, we present a series of alternative uses of structural compliance for the development of simple, adaptive, compliant and/or under-actuated robot grippers and hands that can efficiently and robustly execute a variety of grasping and dexterous, in-hand manipulation tasks. The proposed designs utilize only one actuator per finger to control multiple degrees of freedom and they retain the superior grasping capabilities of the adaptive grasping mechanisms even under significant object pose or other environmental uncertainties. More specifically, in this work, we introduce, discuss, and evaluate: (a) a design of pre-shaped, compliant robot fingers that adapts/conforms to the object geometry, (b) a hyper-adaptive finger-pad design that maximizes the area of the contact patches between the hand and the object, maximizing also grasp stability, and (c) a design that executes compliance adjustable manipulation tasks that can be predetermined by tuning the in-series compliance of the tendon routing system and by appropriately selecting the imposed tendon loads. The grippers are experimentally tested and their efficiency is validated using three different types of tests: (i) grasping tests that involve different everyday objects, (ii) grasp quality tests that estimate the contact area between the grippers and the objects grasped, and (iii) dexterous, in-hand manipulation experiments to evaluate the manipulation capabilities of the Compliance Adjustable Manipulation (CAM) hand. The devices employ mechanical adaptability to facilitate and simplify the efficient execution of robust grasping and dexterous, in-hand manipulation tasks.

## 1. Introduction

Robotic end-effectors have evolved over the past few decades from simple, parallel jaw grippers to dexterous hands that require complicated control laws and sophisticated sensing. The control of such devices is typically computationally expensive when performing versatile object manipulation and grasping (Ma et al., 2013; Odhner et al., 2014). By introducing elastic elements into traditional robotic structures, the successful execution of robust grasping tasks under object pose uncertainties in unstructured environments can be achieved (Odhner and Dollar, 2011; Kim et al., 2013). This added elasticity, or structural compliance, is a key characteristic that increases grasp stability and conformability of the gripper/hand to various object shapes. Early research focused on creating flexible parallel jaw grippers that could conform to various objects (Schmidt, 1978), and more recent research explores applications outside of industrial settings that involve interactions with soft, biological materials (Tai et al., 2016). Structural compliance increases grasping robustness, allowing end-effectors to deal with uncertainties in object positioning and surface geometries (Liarokapis and Dollar, 2017), and increases contact friction through compliance matching (Majidi, 2014).

Object stability within the gripper is usually maintained by the friction force between the gripper surface and the object during grasping. In order to increase this friction force without increasing the applied gripping force, structurally compliant grippers could exploit an increase of the contact area and use surface materials with high friction coefficients to provide better non-permanent adhesion between the object and the gripper. The added elasticity facilitates the accommodation of uncertainties and errors in object and hand positioning, which is of paramount importance when interacting with unstructured environments (Niehues et al., 2015; Liarokapis and Dollar, 2018).

Alternatively, structural compliance could also be used for in-hand manipulation. Traditionally, tendon driven underactuated systems have rigidly anchored tendons, and any attempt at increasing the tendon tension upon object contact would result in vast finger reconfiguration (change of finger configuration/pose). However, the use of non-rigidly anchored tendons (in-series compliance) could facilitate the actuation of other mechanisms such as rotating finger pads or fingernail extensions for in hand manipulation or enhancement of the grasping capacities. The compliance based mechanical adjustment of the motion of these mechanisms depends on the stiffness of the joints.

Over the last decades, various designs of adaptive grippers have been proposed that facilitate the execution of robust grasping and dexterous manipulation tasks. These designs exhibit some form of structural compliance, and most of them are also underactuated. An underactuated design provides simplicity in operation and control and significantly reduces development cost as the number of motors is minimized. Significant research effort has also been put into investigating alternative hand geometries and kinematics, which led to non-conventional hand designs. There have also been significant effort in making those hands freely available, using open-source dissemination and providing adequate documentation for design replication (Zisimatos et al., 2014; Kontoudis et al., 2015; Ma and Dollar, 2017).

**Figure 1:**
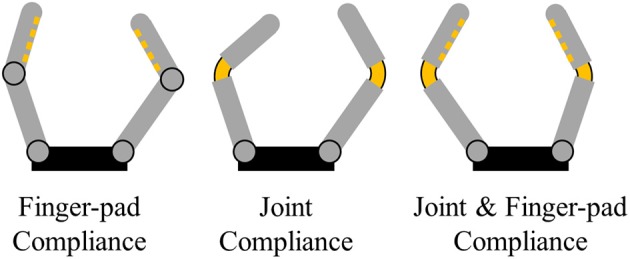
Typical uses of structural compliance in adaptive robot grippers and hands: Finger-pad compliance, joint compliance and combined joint, and finger-pad compliance.

Design approaches to implement structural compliance can roughly be categorized into two major approaches: adaptive, tendon-driven mechanisms employing structural compliance in the joints and finger-pads levels and soft robotic mechanisms using fully compliant structures and pneumatic or hydraulic actuation paradigms. Designs such as the Yale Open Hand project devices (Ma et al., 2013) use fingers with multiple elastic joints and soft finger-pads to increase their gripping capabilities and conformability to the shapes of the grasped objects. Other designs such as the robot gripper from Robotiq's adaptive gripper range (Robotiq, 2019a,b) or Festo's adaptive finger gripper (Festo Coorporate, 2019) employed series elastic differential transmissions. Highly structural compliant soft continuum grippers like the Versaball (Empire Robotics, 2019) from Empire Robotics, Ocean One's soft grippers (Stuart et al., 2017) or Soft Robotics's soft gripper (Robotics, 2019) are capable of grasping objects of various geometries by conforming to the objects exterior hence increasing the number of contact points. Limitations of such designs are usually observed when manipulating very small or very soft objects where the membrane cannot form a stable contact (Brown et al., 2010). This trade-off between soft and rigid grippers outlined by Hughes et al. describes the relationships between precision, structural compliance, DOF, and force exertion (Hughes et al., 2016). Soft and continuum body manipulators benefit from high DOF and large deformation capacities. Comparatively, adaptive, tendon-driven mechanisms are more robust, have better force exertion capabilities, and achieve higher precision. Traditionally, for the creation of adaptive, tendon driven hands, structural compliance is introduced either in the joints (e.g., flexure joints) (Dollar and Howe, 2006; Odhner et al., 2014) or in their finger-pads (Shimoga and Goldenberg, 1996; Carpenter, 2014), increasing the mechanical adaptability and contact surface compliance of the overall grasping mechanism (Figure 1). Joint compliance in underactuated designs allows grippers to grasp objects with unknown object poses using minimalistic control schemes and reduced number of actuators.

In this paper, we present alternative uses of structural compliance for the development of adaptive robot grippers and hands and we evaluate the performance trade-offs when using such design approaches for robust grasping and dexterous manipulation. More precisely, we propose a gripper that executes compliance adjustable manipulation motions and two different adaptive robot grippers with pre-shaped fingers and hyper-adaptive finger-pads. Both designs were developed in order to maximize the area of contact patches between the fingers and the grasped objects. This also maximizes specific grasp quality measures, extracting more robust and stable grasps. The hyper-adaptive finger-pads rely on a pin array design, similar to the design presented by (Flintoff et al., 2018; Mo et al., 2018). With these simple elastic modules, high deformation and ability to conform to the object shape are achieved. Furthermore, due to the continuum elastic behavior of these padded surfaces, object reconfiguration or slippage may occur during grasping or manipulation. Also, for irregularly shaped objects, the contact surface deforms in a non-uniform manner. The multi-material pre-shaped finger design relies on the combination of various elastic elements. The polyurethane core provides a stiffer backbone that increases force transmission while the pre-shaped curvature enhances the ability of the finger to conform to the object geometry. The silicone skin increases the surface friction coefficient and gripping when grasping everyday life objects. The pre-shaped finger design aims to increase the total area of the contact patches during grasping. Regarding dexterous, in-hand manipulation, we propose an adaptive robot hand that takes advantage of compliance adjustable manipulation motions. The hand can be equipped with rotation and translation modules on the distal phalanges of the fingers that facilitate the execution of local manipulation motion. The timing of the triggering of the manipulation motions depend on the stiffness of the joints and is facilitated by the introduction of in-series compliant elements in the tendon routing system. All the proposed robot hand and gripper designs are underactuated and of minimal cost, weight and complexity. The efficiency of the proposed mechanisms is experimentally validated with a variety of experiments involving robust grasping with everyday life objects. All designs will be made publicly available (in an open-source manner) to facilitate replication by other research groups.

The rest of the document is organized as follows: section 2 presents the employed grasp quality measures, section 3 focuses on a series of alternative uses of structural compliance and designs of adaptive robot hands, section 4 presents the experimental procedures, section 5 presents and discusses the results, while Section 6 concludes the paper and discusses possible future directions. A list of abbreviations and acronyms used throughout the paper is provided in Table 1.

**Table 1:** List of acronyms and abbreviations.

CAM	Compliance Adjustable Manipulation
GWS	Grasp Wrench Space
HA	Hyper Adaptive
P-HA	Parallel Jaw Hyper Adaptive
HDM	Hybrid Deposition Manufacturing
MS-PSA	Multi-Segmented Core Pre-Shaped Adaptive
PSA	Pre-Shaped Adaptive
PDE	Partial Differential Equations
YCB	Yale-CMU-Berkeley


## 2. Grasp Quality Measures

Task execution with a robotic hand is primarily dependent on the robust grasping of an object, which can be defined as the hand's ability to constrain the object motion. An effective grasp is characterized by the ability of the hand to withstand external disturbances while maintaining stable object contact. In general, a hand can grasp a given object in multiple ways. Quantifying the grasp quality is essential for the optimization and selection of appropriate grasp types. In this study, we use the Grasp Wrench Space quality measure to quantify and visualize the effects of increased size of the contact patches on the effectiveness of the grasp.

**Figure 2:**
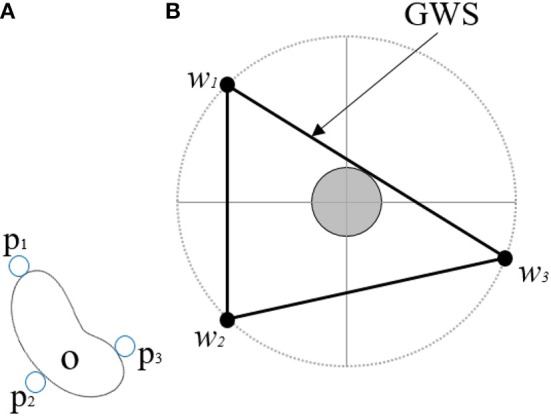
**(A)** Depicts an object *O* being grasped at contact points *p*_1_, *p*_2_, *p*_3_ using a point contact model. **(B)** Presents the Grasp Wrench Space (*GWS*) generated by this grasp *G* and wrenches *w*_1_, *w*_2_, *w*_3_.

The torques applied at each one of the joints generate a finger force *f*_*i*_ at the fingertip *i*. The force *f*_*i*_ applied on the object at a point *p*_*i*_ generates a torque τ_*i*_ with respect to the object's center of mass. A wrench vector ω_*i*_ is the combination of these force and torque components defined as ω_*i*_ = (*f*_*i*_, τ_*i*_/ρ), where ρ is a constant used to define the metric of the wrench space (Roa and Suárez, 2009). A grasp *G* is defined as the set of all the points on the object surface that are in contact with the fingers. Consider an object *O* as shown in Figure 2 that is being grasped by fingertips at the points *p*_1_, *p*_2_, *p*_3_. A point contact model provides the forces and twists acting at each of these points. We adopt Coulomb's friction model by approximating the friction cone at the contact point *p*_*i*_ by a pyramid with *m* edges. The finger force *f*_*i*_ exerted by the finger *i* at this point can be expressed as a linear combination of primitive forces *f*_*ij*_, *j* = 1, …, *m* along the pyramid edges and wrench *w*_*i*_ produced by *f*_*i*_ at *p*_*i*_ can be expressed as a positive linear combination of primitive wrenches *w*_*ij*_. The resultant wrench produced by the *n* fingers can be calculated as,

(1) $$\begin{array}{l} W\left[G\right]=\overset{n}{\underset{i=1}{\sum }}\omega_{i}=\overset{n}{\underset{i=1}{\sum }}\overset{m}{\underset{j=1}{\sum }}\alpha_{ij}\omega_{ij}\\ \;with\;\alpha_{ij}\geq 0,\overset{n}{\underset{i=1}{\sum }}\alpha_{ij}\leq 1 \end{array}$$

where *W*[*G*] denotes the set of all wrenches associated with the contact points of grasp *G*. The set of all the wrenches that can be applied to the object through the grasp *G* is called the Grasp Wrench Space (GWS). GWS is defined as the convex hull of the primitive wrenches associated to the contact points in *G*

(2) $$\begin{array}{r} GWS=ConvexHull\left(W\left[G\right]\right) \end{array}$$

**Figure 3:**
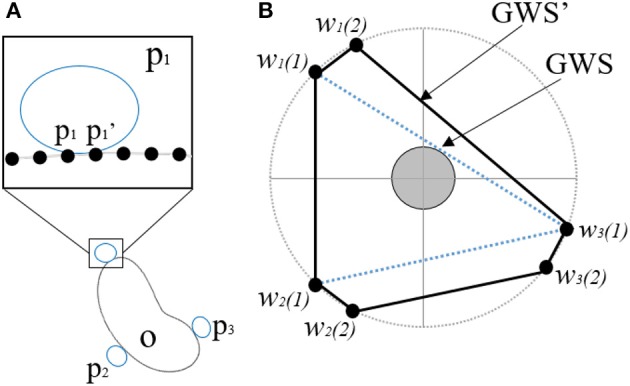
**(A)** Presents an object *O* being grasped at contact points *p*_1_, *p*_2_, *p*_3_ using a patch contact model. The inset figure shows the additional points with-in the patch that are being included, while **(B)** demonstrates that the grasp wrench space *GWS*′ generated by the contact patch with additional points is significantly higher than the *GWS* generated by the initial points.

The GWS can be described as the largest perturbation wrench the grasp can resist in any direction (Roa and Suárez, 2014). The higher the volume of this grasp wrench space, the better the grasp. In order to visualize the effect of increased size of the contact patches provided by the hyper adaptive fingers defined in this paper, we calculate the GWS of patch contacts instead of the point contact model (Charusta et al., 2012). If the hand makes a patch contact centered at point *p*_*i*_, the patch is defined as

(3) $$\begin{array}{r} P\left(i,r\right)=\left\{z\;:\;\delta_{i}^{z}\leq r,z\in O\right\} \end{array}$$

where *r* ≥ 0 is the parameter that bounds the size of the patch and $\delta_{i}^{z}$ is the shortest edge between the points with indices *i* and *z*. This means that the point *p*_*z*_ qualifies to be a member of a patch around *p*_*i*_ if the distance between *p*_*i*_ and *p*_*z*_ is less than or equal to *r*. The physical significance of this adapted quality measure is that a bigger contact patch would provide a higher number of contact points, thereby significantly increasing the wrenches exerted on the object and grasp quality (stability of grasp). Figure 3 demonstrates the effect of the additional wrenches exerted on the volume of the Grasp Wrench space. The new wrench space *GWS*′ formed using patch contact is a superset of the GWS formed by the single point contacts. The compliance of the hands discussed in this paper allows them to conform to the shape of the objects being grasped thereby generating very large contact patches and increased GWS. This ensures the stability of the grasp and its ability to resist disturbances.

## 3. Alternative Designs and Uses for Structural Compliance

In this section, we introduce three different designs employing alternative uses of structural compliance for the development of adaptive robot hands.

### 3.1. Pre-shaped Adaptive Robot Fingers

**Figure 4:**
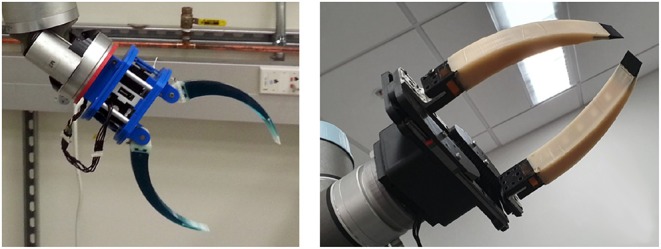
Example of a gripper equipped with single core Pre-Shaped Adaptive (PSA) robot fingers with different curvatures and rigid fingernails embedded in the elastomer material **(Left)**. Example of a parallel jaw gripper equipped with Multi-Segmented core Pre-Shaped Adaptive (MS-PSA) fingers with rubber skin and L shaped mounting bases **(Right)**.

**Figure 5:**
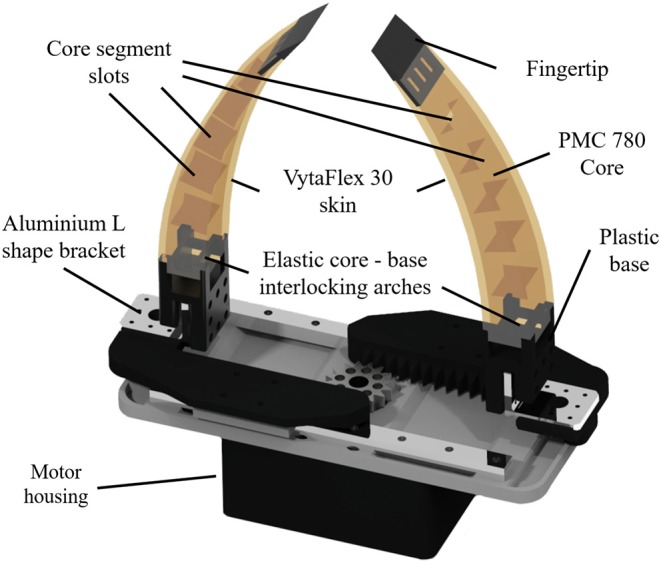
3D model of a parallel jaw gripper equipped with two MS-PSA fingers. The Multi-Segmented core Pre-Shaped Adaptive (MS-PSA) finger is composed of an inner segmented core, exterior silicone skin that has good gripping properties (high friction with plastic), a plastic fingertip, and a base. The segmented core provides anchors for the skin layer material to take the desired shape and prevents layer peeling. The base and the fingertip have appropriate hollowed arches that provide mounting points for the elastic elements, using the concept of Hybrid Deposition Manufacturing (HDM) (Ma et al., 2015). The main source of force transmission and compliance comes from the segmented core while the skin provides a higher friction coefficient with plastic when grasping.

The Pre-Shaped Adaptive (PSA) finger is an elastic, compliant robot finger designed for maximizing the contact area between the object and the finger during grasping (see Figure 4). The finger employs a pre-shaped structure that increases its ability to conform around multiple object shapes. After contact with the object surface the finger starts straightening conforming also to the object shape. Two types of PSA fingers were developed, a single core PSA finger and a multi-segmented core version (MS-PSA), as shown in Figure 5. Both fingers consist of a polyurethane rubber (Smooth-On PMC-780) core with a Polylactic Acid (PLA) fingernail and a mounting base. To increase surface friction and the compliance of the finger, the MS-PSA finger is covered by a layer of Vytaflex-30. The five cavities in the polyurethane rubber core provide anchors for the urethane rubber (Smooth-On Vytaflex-30) skin and act as segmented regions with different elastic properties during reconfiguration for the MS-PSA finger.

**Figure 6:**
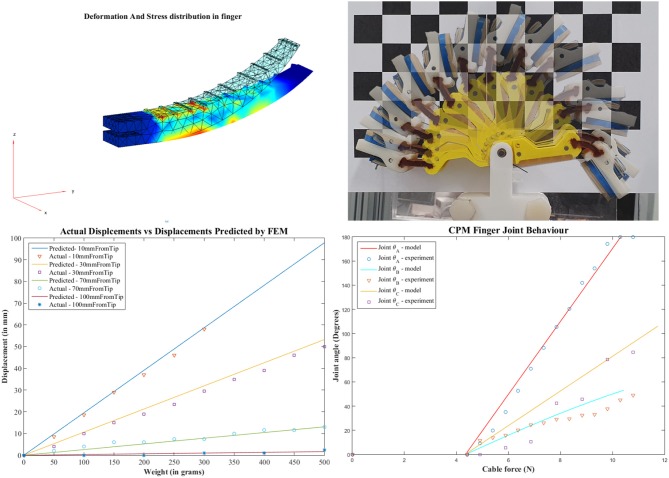
FEM analysis of a PSA finger **(Left top)** and simulated vs. measured structural deformation **(Left bottom)**. Weights were mounted at 10, 30, 70, and 100 mm from the tip and the tip displacement was recorded. Time lapse of CAM finger motion **(Right top)** and joint behavior **(Right bottom)**. Model simulated cable forces had to overcome a friction force of 4.905 N before finger motion occurred.

The reconfiguration of the finger allows it to also conform to rectangular and non-round objects according to the forces exerted on their surface. Notably, PSA robot fingers cannot fully resist shear forces as they experience out of plane motions during grasping. Also, for small objects, grasping is commonly performed near the rigid fingertips where the grasp force is more dependant on the elastic properties of the fingers. Depending on the object parameters, during fingertip grasping or pinching, the object may reconfigure into the most elastic regions of the finger. It was more reliable to grasp objects within the elastic regions of the PSA finger to allow the finger to conform to the object geometry. The PSA finger was mounted with a pivoting base to increase the grasp area. The MS-PSA model was mounted on a rigid base onto the parallel jaw gripper. Pre-shaped adaptive robot fingers can be easily controlled since the finger does not have multiple joints and phalanges, relying on a single motor for actuation. For the modeling of the preshaped finger, the behavior is similar to a curved and tapered cantilever beam. The effects of various simulated loads on different parts of the finger are simulated using finite element analysis. We used Matlab's Partial Differential Equations (PDE) Toolbox™ to perform the finite element analysis and analyze the structural mechanics of the finger assuming single material. A 3D model of the finger is imported to Matlab and associated with a PDE model object, the PDE toolbox recognizes the various surfaces of the object and marks them as faces. All the faces associated with the base of the finger used to mount it on the robotic hand are set to “Fixed” cantilevering the fingers. The Young's modulus (*E*) of PMC 780 Wet is 400 psi, and a Poisson's ratio (*v*) of 0.49995 is set for the compliant section of the finger structure. The magnitude and face (surface) of the load (*f* = body force) are specified as boundary conditions. The toolbox then generates a tetrahedron mesh of the finger consisting of 2,211 nodes and 8,676 elements. The partial differential equations are solved for the nodes to provide the effective stress, displacement, and deformation. Figure 6 compares the displacement predicted by the FEM model against the actual displacements measured on the finger when incremental loads are applied at various positions on the finger surface.

For a given stress σ, body force (*f*), strain (ϵ), and displacement *u*, the equilibrium equation is given by

(4) $$\begin{array}{r} -\nabla .\sigma =f \end{array}$$

The toolbox form of the equation for the given 3D problem is given by

(5) $$\begin{array}{r} -\nabla .\left(c⊗\nabla u\right)=f, \end{array}$$

and the strain-displacement relationships is

(6) $$\begin{array}{r} \epsilon =\frac{1}{2}\left(\nabla u+\nabla u^{T}\right) \end{array}$$

Equations (4)–(6) are solved for each node in the mesh to provide the resultant displacement of the finger and Von Mises stress, as shown in Figure 6.

The forces can only be applied to the faces recognized by the PDE toolbox. However, when the 3D file is imported, the toolbox ignores the faces separated by small angles and merges them as a single face. This limits us from applying forces to various sections of the finger separately as the entire top of the finger is recognized as a single face. In order to overcome this limitation, we have added ridges to the area of the finger model we need to apply the forces to create faces recognizable by the toolbox.

### 3.2. Hyper Adaptive Finger

**Figure 7:**
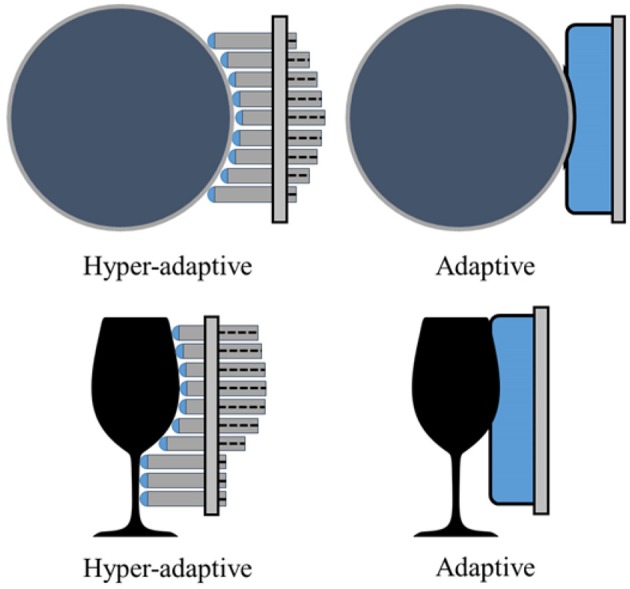
A comparison between the hyper-adaptive finger-pad design/concept and a classic adaptive finger-pad with increased compliance. The classic design (e.g., finger-pad employed by Ma and Dollar, 2017) exhibits local deformability while the hyper-adaptive paradigm conforms to the exact object shape. The hyper adaptive design reduces the shear stress and slippage of the object grasped due to independent contact regions between the object and the finger-pad.

**Figure 8:**
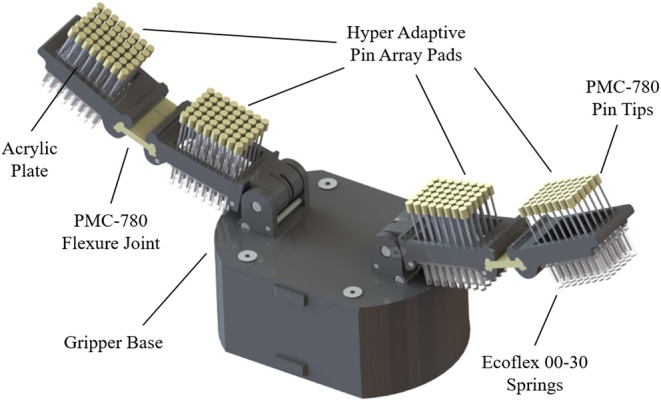
The Hyper Adaptive (HA) finger consists of pin array pads (hyper-adaptive finger-pads), acrylic plates, polymer springs, and plastic phalanges that support the pads. The pin array pads consist of 48 pins each. Each pin has a compliant rubbery tip to increase the friction between the tip and the object. The pins are mounted onto a 10 mm thick acrylic guide plate that is connected to the plastic phalanges. The compliance of the finger pads allows each finger to reconfigure to the exact object shape. The HA finger distributes the contact forces to each pin, ensuring the stability and robustness of the grasps executed.

Similarly to the pre-shaped robot fingers concept, the motivation for the development of the hyper-adaptive finger-pads comes from the desired maximization of the contact areas between the hand finger-pads and the object surface. This concept uses adaptive micro-structures that conform to the object's geometry in a “divide and conquer” manner and constrains the object inside the grasp. The distributed forces across the finger pad during the grasp reconfiguration ensure a stable grasp, as shown in Figure 7. It must be noted that the hyper-adaptive finger-pads are compliant only in one direction, and thus they resist shear forces. This differs from traditional, compliant structures that deform equally in all directions, such as foam, silicones, and other soft materials. The Hyper Adaptive (HA) finger, shown in Figure 8, is composed of pin array pads, acrylic plates, polymer springs, and plastic phalanges. The pin array pads consist of 48 pins (6 × 8 array) of 1.1 mm diameter made out of steel (each finger has two pin arrays). Each pin has a compliant rubbery tip made out of Smooth-On PMC-780 that increases the friction between the finger and the object. The pins are mounted onto a 10 mm thick acrylic guide plate that is connected to the plastic phalanges. The acrylic plate is used to maintain a tight tolerance between the plate and the pins, guaranteeing stable and unidirectional motion. In order to reduce the weight and complexity of the system, traditional return springs were replaced by an elastic polymer tube array made of Smooth-On Ecoflex 00-30. This design choice also reduces the final cost and weight of the hyper adaptive finger. The compliance of the finger pads allows the finger to reconfigure to the object shape. Doing so, the adaptive finger distributes the contact forces to each pin, ensuring stability and robustness at each grasp executed. The hyper adaptive fingers use a torsion spring at the pin joint and a flexure joint (made out of Smooth-On PMC-780) between the two phalanges of each finger. The finger pads and the fingers were designed to be easily replaced if a different base or mount is needed. In the experiments, the gripper was tested using two different bases: a base adapted from the Yale Open Hand Model T42 (Ma and Dollar, 2017) and a base of a parallel jaw gripper. The Hyper-adaptive fingers are controlled similarly to the traditional adaptive robot fingers, with the advantage of more stable grasp, as they increase the contact area between object and finger.

The behavior of the pin array design that is used on the hyper adaptive fingers can be described using parallel coupled springs. Each pin receives the grasping force from the fingers resulting in different levels of deformation. The force is distributed among the contact pins.The force exerted by an individual pin is given by,

(7) $$\begin{array}{r} f_{i}=K\Delta x \end{array}$$

where K is the spring constant, and Δ*x* is the change in length of the compliant cover of each pin.

The spring constant K is the result of the elastic modulus *y* of Smooth-On Ecoflex 00-30. It can be calculated as

(8) $$\begin{array}{r} K=YA/L \end{array}$$

where Y is 10 psi, and it is the elastic modulus of Ecoflex, L is 2 mm and is the length of Ecoflex layer and A is 12.57 *mm*^2^, and is the area of the pin head. As the pins act as parallel springs, the effective spring constant can be calculated by

(9) $$\begin{array}{r} K=k_{1}+k_{2}+\ldots +k_{n} \end{array}$$

The effective force exerted is further increased by the PMC 780 coating on the grasp surfaces which increases the friction force as follows

(10) $$\begin{array}{r} F_{f}=\mu F_{g} \end{array}$$

where, *F*_*f*_ is the frictional force, μ is the co-efficient of friction of PMC 780, and *F*_*g*_ is the gripping force exerted by the fingers. The gripping force *F*_*g*_ required to grasp a given object of mass M is provided by the equation

(11) $$\begin{array}{r} F_{g}=\frac{MgS_{F}}{2\mu } \end{array}$$

where μ is the co-efficient of friction and *S*_*F*_ is the safety factor.

### 3.3. Compliance Based Adjusting of Grasping and Manipulation Motions

**Figure 9:**
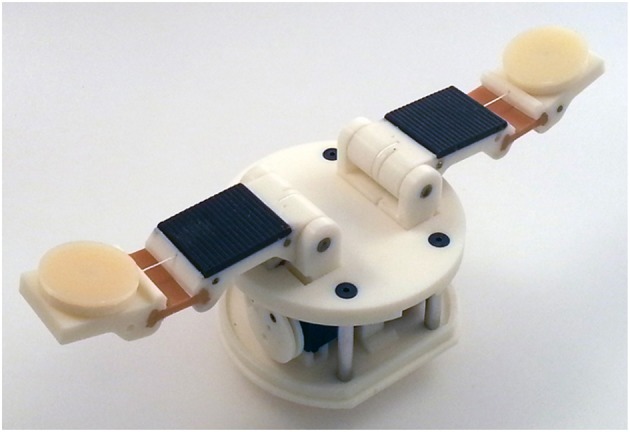
A two-fingered Compliance Adjustable Manipulation (CAM) adaptive hand with a rotation module per fingertip. The development of the hand is based on the Hybrid Deposition Manufacturing (HDM) technique (Ma et al., 2015). The base of the hand is the base of model T42 of the Yale OpenHand project (Ma and Dollar, 2017).

**Figure 10:**
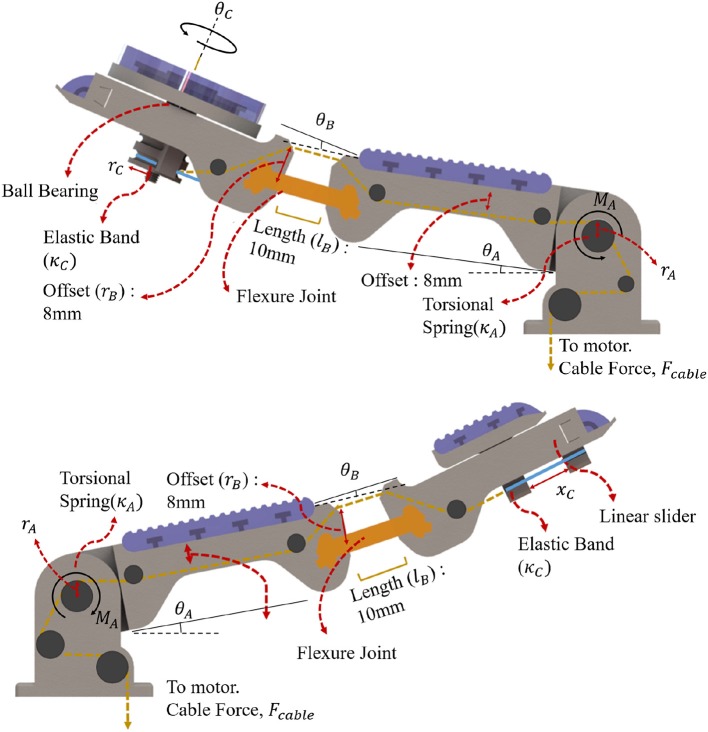
Finger structures of the Compliance Adjustable Manipulation (CAM) gripper for the case of an extra rotation and translation degree of freedom located at the fingertip. An elastic band is connected in series with the tendon routing for both cases. For the extra rotation finger **(Top)**, the elastic band is wrapped around a pulley connected with the ball bearing and the rotating part. When the tendon is tensioned, the finger closes until it touches the object. Then, the tendon tension increases, unwrapping it around the pulley connected to the rotation module, and promoting rotation of the object grasped. For the extra translation finger **(Bottom)** the elastic band connects the moving part (that moves along appropriate slides) with the fingertip. When the tendon is tensioned, the finger closes until it touches the object. Then, the tendon tension increases, pulling the translation module at the distal phalanx, sliding the object grasped.

The concept of compliance based adjustable motions focuses on introducing in-series compliance in the tendon routing system (see Figure 9) that facilitates the execution of dexterous, in-hand manipulation tasks. The CAM gripper design allows us to execute both grasping tasks (through simple finger flexion) and dexterous, in-hand manipulation tasks employing a single actuator per finger (for both cases). This was done through the appropriate displacement of an extra DOF the motion of which is affected by the tuning of the in-series compliance. It must be noted that a careful selection of the joint stiffness and the in-series compliant elements can change the tendon loads required to trigger the grasping and the manipulation motions (Figure 10) and the timing of their triggering. Thus, the particular concept allows us to pre-adjust the hand motions by selecting the stiffness values of the compliant elements. The extra DOFs can facilitate the execution of a variety of dexterous manipulation tasks (e.g., in-hand manipulation, extrinsic dexterity tasks, flip-n-pinch, etc.). More details can be found in Figures 9, **14**.

The CAM gripper proposed has an extra rotation and translation degree of freedom located at the fingertip. An elastic band is connected in series with the tendon routing for both cases. For the rotation module, the elastic band is wrapped around a pulley connected with the ball bearing and the rotating part. When the tendon is tensioned, the finger closes until it touches the object. Then, the tendon tension increases, unwrapping it around the pulley connected to the rotation module, promoting rotation of the object grasped. For the translation module, the elastic band connects the moving part (that moves along appropriate slides) with the fingertip. When the tendon is tensioned, the finger closes until it touches the object. Then, the tendon tension increases, pulling the translation module at the distal phalanx, sliding the object grasped. The behavior of CAM gripper is determined by a torsional spring, an elastic flexure joint, and a elastic element loaded linear-rotational joint (extra DoF). The base of the finger is loaded by a torsional spring, by taking the sum of moment about each joint:

(12) $$\begin{array}{r} \sum M_{A}=\tau_{cable}-\tau_{spring} \end{array}$$

where the cable tension moment τ_*cable*_ must be greater than τ_*cable*_ for the finger to initiate grasping motion. The expected angle from given cable tension is given by:

(13) $$\begin{array}{r} \Delta \theta_{A}=\frac{r_{a}\left(F_{cable}-F_{friction}\right)}{k_{a}} \end{array}$$

where θ_*A*_ is the amount of rotation of the extra DoF pad, *k*_*a*_ is the elastic constant of the torsional spring and *r*_*a*_ is the radius or distance between the cable mount and the center of the joint. The second joint is composed of a flexure joint and can be approximated by the smooth curvature model described by Odhner and Dollar (2012). The joint behavior is given by:

(14) $$\begin{array}{r} \frac{\Delta \theta_{B}}{cos\left(\Delta \theta_{B}/2\right)}=\frac{2l_{b}r_{b}\left(F_{cable}-F_{friction}\right)}{EI} \end{array}$$

where *E* is the Young's modulus of Smooth-On PMC-780, *I* is the second moment of area of the flexure joint, *l*_*b*_ is the length of the flexure joint and *r*_*b*_ is the distance between the flexure joint and cable anchor. The fingertip joint composed of a linear elastic element connected to a pulley with the tendon cable pulling the pulley in the opposite direction. The joint behavior can be described by:

(15) $$\begin{array}{r} \Delta \theta_{C}=\frac{360\left(F_{cable}-F_{friction}\right)}{2\pi k_{c}r_{c}} \end{array}$$

where *k*_*c*_ is the elastic constant of the linear elastic element, *r*_*c*_ is the radius of the pulley. The linear fingertip version performance can be described by Hooke's law. Each joint is connected to the same tendon, hence, each joint will move slightly when cable tension is applied to the tendon. For compliance based adjustment, we select the stiffness of the in hand manipulation joint. As long as the tension of the tendon is less than the required amount to overcome the stiffness, the in hand manipulation DOF moves negligibly compared to the other joints. The in-hand manipulation, therefore, relies on the inhibition of movement in the finger joints. Notably, due to the structure of the tendon routing, there is considerable friction within the underactuated system. The simulated model and measured results for the PSA finger and the CAM finger are presented in Figure 6. For the PSA finger, the weighted masses were mounted on the finger surface at distances 10, 30, 70, and 100 mm from the tip. The fingertip and the contour of the finger were traced onto paper and the displacements were measured at the location of the mass mount. The model can predict the displacement with a residual standard deviation of 1.5 mm. For the rotatory tipped CAM finger, weighted masses were attached to the cable and results were recorded with a camera at a set distance. Masses were added in increments of 50 g and changes in individual joint angle were estimated from the images taken. In Figure 6, the friction was estimated at 4.41±0.49N taken from the experiment. Of the three joints, joint A, joint B and joint C has a residual standard deviation of 1.77 N, 1.44 N, and 2.04 N per, respectively. Errors were due to the friction of the tendon routing. Other sources may include camera lens magnification and diffraction.

## 4. Experiments

In this section, we present the experiments that were conducted in order to validate the efficiency of the proposed concepts and designs.

### 4.1. Grasping Experiments

**Figure 11:**
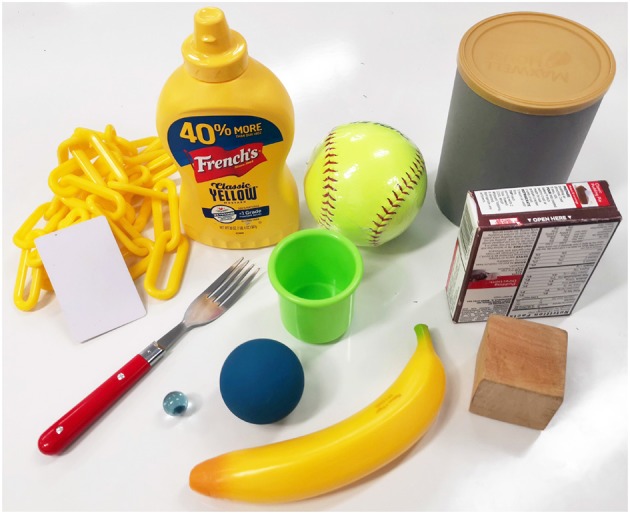
Twelve objects from the YCB object set (Calli et al., 2015) were used in the experiments: a master chef can, a soft ball, a mustard bottle, a chain, a credit card, a fork, a small cup, a jello box, a wooden cube, a plastic banana, a racquetball, and a marble.

**Figure 12:**
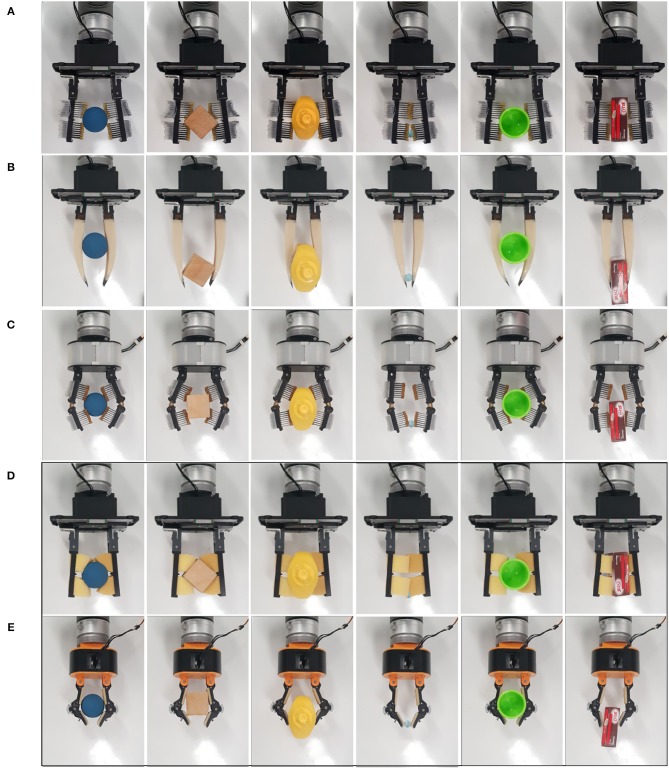
Grasping capabilities comparison of: **(A)** parallel jaw gripper with Hyper-Adaptive fingers (Parallel jaw HA), **(B)** a parallel jaw gripper with Multi-Segmented core Pre-Shaped Adaptive (MS-PSA) fingers, **(C)** a Hyper-Adaptive hand (HA) with fingers based on flexure and spring loaded pin joints, **(D)** a parallel jaw gripper with sponge-like, compliant finger pads, and **(E)** the model T42 of the Yale OpenHand project (Ma and Dollar, 2017). The objects used are: a small ball, a wooden cube, a mustard bottle, a marble, a small cup, and a jello box. All objects are included in the YCB object set (Calli et al., 2015). **(D,E)** (enclosed in a black frame) focus on grippers that are used for comparison purposes.

**Figure 13:**
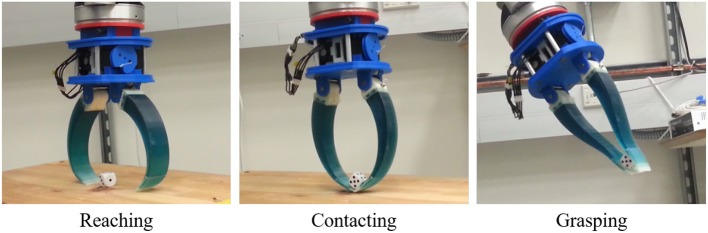
A robot gripper equipped with two Pre-Shaped Adaptive (PSA) robot fingers performing reaching, contacting, and grasping tasks with a dice. Upon contact, the PSA fingers adapt to the object geometry, maximizing the area of the contact patches between the hand and the object surface.

- **Object Grasping:** The first robust grasping experiment focused on evaluating the proposed designs by assessing grasp stability using objects from the Yale-CMU-Berkeley (YCB) object set (Calli et al., 2015). A selection of daily objects shown in Figure 11 was used. Individual objects were placed on a flat surface and the grippers were attached to a robot arm (UR5) for grasping. For each object, the gripper executes a grasp, and the robot arm then lifts the object and hold for 5 s. The object then experiences disturbances from the arm moving repeatedly in the horizontal direction and finally placed back on the surface. Assessment of grasp stability was based on a successful grasp with no visible object reorientation (motion in any direction) or slippage during the task execution. Further grasp stability evaluation following the YCB gripper assessment protocol and benchmark (Calli et al., 2015) was conducted.
  In Figure 12, we present a grasping experiment comparing how different grippers conform around objects. The objects were randomly placed within the grasping range of the grippers. Stable grasps were achieved when the object center of mass was within the grasping range of the grippers over ten trials. The sponge finger demonstrated extreme padding compliance, where large deformation occurs. The lack of shear resistance made lifting heavy objects difficult.
  In Figure 13, we demonstrate how PSA grippers adapt to non-spherical objects. Although the initial shapes of the fingers are optimized for grasping round objects, the PSA fingers were able to reconfigure the finger structures to conform to the dice geometry. Similarly, the MS-PSA gripper could adapt to various object geometries and the additional skin layer provides extra stability for the grasped objects.
- **Contact Area:** The second robust grasping experiment focused on measuring the contact surface area of the grippers using chalk and acrylic paint residues on a layer of paper that was wrapped around the selected objects. The small cup and mustard bottle were chosen over the other objects due to their size, that fits within all grippers. Objects were placed in the same initial position.The contact area was then obtained by measuring the estimated squares that best fitted the sample as shown in **Figure 15**. Surface contact transfers the paint on the grippers onto the paper highlighting the respective contact surface areas. The robot arm raises the gripped object after a stable grasp is achieved and places the object back on the table surface for grasp release. The painted areas of the paper are then measured. Results are reported in **Table 3**.
- **Clench Force:** The maximum clench force was also measured for each gripper. A BioPac SSLA25 dynamometer was placed within the grasping workspace of each gripper and the device was actuated until motor stall. The maximum clench force was recorded for each gripper and estimated from 10 trials. The parallel jaw gripper had a single Dynamixel high torque XM-430 smart motor while the Yale Open Hand Model T42, the HA, and the rotary fingertip module grippers utilize two of these motors. Results are reported in **Table 3**.

### 4.2. Dexterous Manipulation Experiments

**Figure 14:**
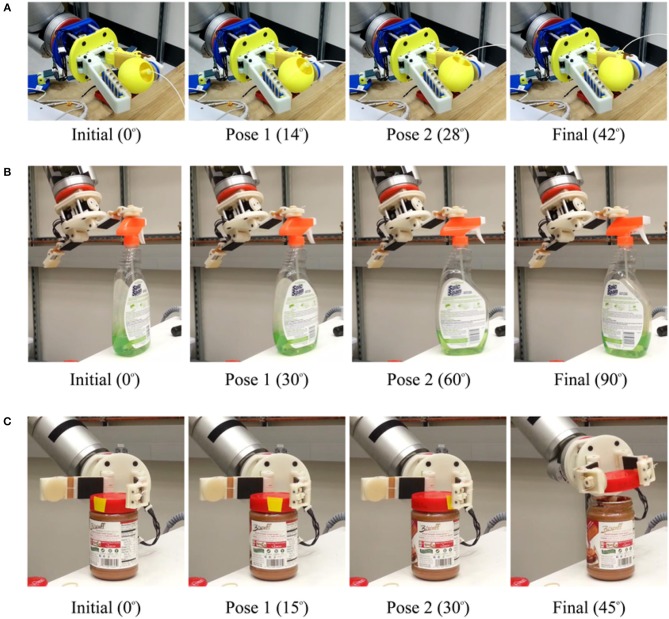
**(A)** presents a dexterous, in-hand manipulation experiment executed with a Compliance Adjustable Manipulation (CAM) hand, equipped with a rotation module in the distal phalanx of the moving finger and a steady thumb. The robot hand performs an in-hand rotation of a 3D printed sphere. **(B)** presents a dexterous, in-hand manipulation experiment conducted with a CAM hand equipped with one translation module and one rotation module. The gripper uses the concept of extrinsic dexterity to rotate a bottle of Windex spray using the table surface and the rotation module of the right finger's distal phalanx. The center of mass of the object was aligned to the finger pads to successfully rotate the object. This is a classic example of how the exploitation of certain environmental constraints may facilitate the execution of manipulation tasks (Dafle et al., 2014). **(C)** presents a dexterous, in-hand manipulation experiment conducted with a CAM hand equipped with one translation and one rotation module. The robot hand uses the translation module of the distal phalanx of the left finger and the proximal phalanx of the right finger to unscrew the lid of a jar bottle.

In this subsection, we evaluate the manipulation capabilities of the CAM hand. The hand can be equipped with a variety of extra DoFs on the fingertips that can execute translational or rotational motions. In Figure 14A, a gripper that has a passive finger (e.g., thumb) and an active finger with an extra DoF that implements a local rotation of the contact was used to grasp a sphere firmly and then to rotate it using the same motor. In Figure 14B, a rotation module is used to rotate a bottle of Windex spray using the concept of extrinsic dexterity (Dafle et al., 2014). In Figure 14C, a translation module is used to unscrew the lid of a jar. In all cases, upon contact with the object surface, the load exerted on the finger motors becomes an equivalent displacement of the extra DoF, executing the corresponding manipulation task. Although the CAM gripper was characterized by a significant post-contact reconfiguration of the extra DoFs, the grasped object remains constrained and the grasping task is executed successfully.

## 5. Results and Discussion

In this section, we present the performance of the hyper adaptive, adaptive and compliance based adjustable designs. The accompanying video presents a comprehensive set of grasping and manipulation tasks executed with the proposed robot grippers. During the experiments, a wide range of everyday life objects was used.

www.newdexterity.org/hyperadaptive

### 5.1. Robust Grasping

**Figure 15:**
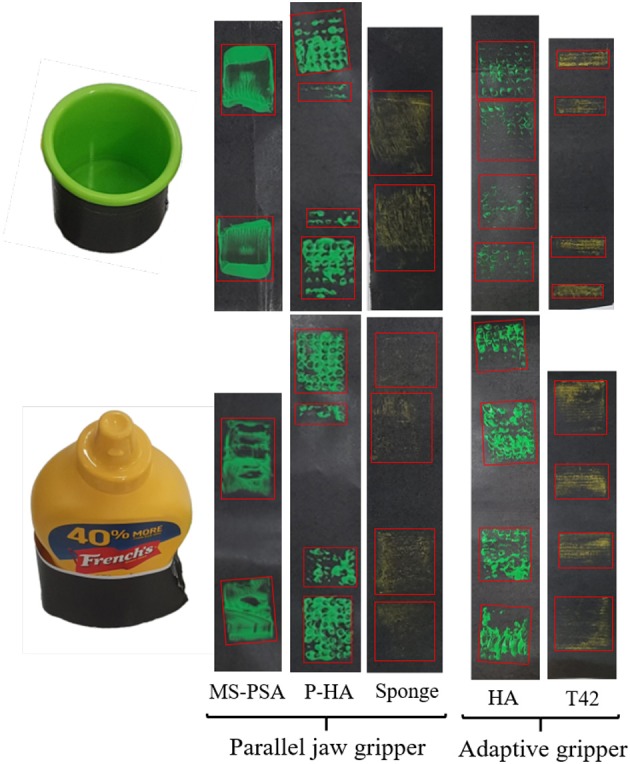
Example of identification and comparison of contact areas for different types of hands and grippers grasping two different objects: a small cup **(Top)** and a bottle of mustard **(Bottom)**. Green acrylic paint was applied to the finger-pads of the examined robot grippers and hands while yellow chalk was applied onto the sponge and T42 gripper finger-pads (maintains better contact). The red boxes enclose the estimated surface areas of the contact patches during grasping. The results demonstrate that for grasping the mustard container, the sponge had the largest surface contact area, followed by HA, T42, P-HA, and MS-PSA gripper. While grasping a smaller and lighter cup, the HA gripper had the largest contact area followed by the parallel jaw HA, parallel sponge, and MS-PSA gripper. T42 had the least contact surface with the object.

- **Object Grasping:** Compliance, in general, increases grasp stability, reduces the required grasping force that should act on the object and increases the ability of a gripper to conform to the shape of the objects being grasped. The compliance also increases the area of the contact patches, increasing also the GWS. This ensures the stability of the grasp and its ability to resist disturbances. In Table 2, we present the results of grasping benchmark on various adaptive fingers in order to evaluate the grasping capabilities and compare them with other adaptive robot hands. The sponge finger and the T42 gripper were included in this study for comparison purposes. With T42 representing traditional soft padding approaches and the sponge as an example of extreme compliance. With excessive compliance, as demonstrated in Table 2, the sponge finger failed to provide a stable grasp on objects that are heavy and had small contact areas. The loss of resistance to shear forces requires higher force exertion. Reduced force transmission also increases the gripper profile and reduced grasping workspace.
  The HA and PSA designs focused on finger pad compliance and joint compliance, respectively. Comparing T42, HA and sponge finger pad compliant solutions, the HA mechanism allowed for decoupled surface geometry conformity. Typical finger pads are made of singular elastic materials which have local coupled area deformation. The matrix of pins conforms to largely irregular shapes and provides shear resistance. Furthermore, unused pins provide perpendicular support when in contact with objects, increasing object stability.
  Of the proposed grippers, the MS-PSA scored the highest in the YCB benchmark. This gripper was able to provide a stable grasp for a large range of object shapes and sizes. It lacked the ability to pickup the flat laid hammer securely due to the loss of clench force further away from the finger base. The YCB benchmark awards points for grasping flat, spherical, and irregular shape objects from a flat surface. The HA and P-HA grippers failed to pick up any of the flat objects. For this reason, they have a significantly lower score due to a lack of fingernail design (grasps of flat objects represent 96 out of 404, or 23.8% of the total YCB score).
- **Contact Area:** Results on surface contact area showed that for grasping the mustard container, the sponge had the largest conformity and surface contact area, followed by HA, T42, parallel jaw HA(P-HA), and the MS-PSA gripper. While grasping a smaller and lighter cup, the HA gripper had the largest contact area followed by the P-HA, parallel sponge, MS-PSA gripper, and the T42. From Figure 15, for the parallel jaw HA and HA grippers, the contact area was estimated using the red boundaries. The actual contact area would be less than the estimated area due to the gaps between the pin pad.
  The PSA design focused on joint compliance while the MS-PSA incorporated surface compliant concepts. Pre-shaped design mitigates partially the problems faced with traditional, flat finger pads. Without any rigid structure within the bodies, force transmission depended on the material's elastic modulus. Non-uniform curvature in the PSA design reduces weight and variant compliance depending on object contact position. Similarly, shear resistant and finger robustness are dependants on finger geometry and material. The segmented core and added skin in the MS-PSA design provided more options for PSA fingers. Near the fingertip of PSA fingers, high compliance results in loss of grasp stability. Rubber skins provided extra friction to the finger while the segmented core was intended to decouple local structural reconfiguration. No visible difference in deformation was observed between PSA and MS-PSA grippers. However, the additional rubber skin provided higher shear resistance allowing heavier objects to be lifted with less deformation. Two types of gripper base were used in this paper, a simple parallel gripper and a hyper adaptive gripper. Parallel jaw grippers have a smaller grasp area compared to a similar sized hyper adaptive gripper. However, due to cable tension and gripper mechanism geometries, the parallel jaw gripper was able to exert more force to the fingers than the hyper adaptive gripper. The HA mechanism was considerably more complex to manufacture and assemble. Also, for both P-HA and HA finger grippers, there were no fingertips designed for picking up a small or flat object. This lead to failure in performing grasp on the credit card blank for both grippers. The MS-PSA finger was slightly more complicated to manufacture due to the two-part process of molding. PSA fingers were made from a single mold but adhesion between PMC 780 and the 3D printed PLA parts was weak. Even with the reinforcing arch structures introduced in the MS-PSA design, fatigue and wear were observed near the thin connective sections which may pose a source for structural failure. Unlike traditional pinned joints, PSA fingers rely on the elastic material tensile strength to maintain structural integrity. However, MS-PSA finger's exterior skin provides extra thickness and was more durable than the initial PSA design.
- **Clench Force:** The maximum clench forces were all measured near the base of the fingers. The results are shown in Table 3. The parallel gripper delivered a much higher gripping force than the hyper adaptive gripper. Due to cable routing in the hyper adaptive gripper, clench force is limited by cable friction and gripper geometry. The parallel sponge had the least base clenching force. Highly compliant sponge compression during grasping potentially increased the parallel structure deformation. This was similar to grasping a larger, heavier object, the contact surface vastly increased. Overall, the parallel gripper was capable of exerting between 31 N to 28 N with the fitted fingers. For small cup, the HA provided the largest contact surface followed by the P-HA and sponge. For the heavier and larger flat surfaced mustard container, the sponge had the largest contact surface followed by HA and T42. Considering the 9 N provided by the HA gripper and 12 N by the T42, we can compare the performance of these grippers with Table 2. With the lowest clenching force, the HA gripper was able to successfully grasp and maintain stability on most objects. With the exception of credit card blank, which was due to missing a fingernail design. Similarly, the P-HA gripper was unable to pick up the card due to fingertip design and frame clearance with the pin pads. Also, the limited grasp workspace of the parallel grippers constrained their capability to grasp large objects. The MS-PSA gripper was able to grasp all selected objects but operated with a higher force exertion capability. These observations support the trend where increased compliance decreases required grasping force.
- **Summary of Design Considerations:** Table 4 presents the comparison of some of the gripper specifications. The CAM was the lightest finger when compared with MS-PSA and the heavier HA mechanisms. However, the lightest finger was the parallel jaw with sponge padding. The MS-PSA had the longest fingers out of the fingers compared. Fingers based on the T42 had relatively short overall finger length compared to the MS-PSA. Notably, this length was measured from the fully extended position for all fingers and the pre-shaped finger's curvature provided extra length. HA and CAM had different phalanges lengths to the T42. HA has a longer total finger reach of around 135 mm while the T42 and CAM have around 110mm. Combining these information with results from Tables 2, 3, the HA mechanism, while being slightly heavier, was able to provide a higher contact surface and conformity when grasping objects. It is hard for the HA grippers to pick up small objects such as credit cards due to the lack of a finger nail design. However, with the lighter, lower conformity and smaller contact surface MS-PSA gripper, objects such as the credit card could be picked up with the embedded fingernail design. Furthermore, the CAM finger design was able to provide an extra DOF for in-hand manipulations without increasing the weight of the finger. In general, the proposed underactuated designs demonstrate that the use of structural compliance reduces the number of required motors to achieve precise and stable grasps. Consequently, the cost of the final device is reduced as well as the complexity to control the robot hands.

**Table 2:** Grasp stability results comparison. Stability is assessed as the ability of the hand/gripper to retain a stable grasp during the execution of an arm trajectory that introduces significant disturbances (as seen in the video).

**YCB objects**	**Grippers**									
	**Parallel jaw gripper**						**Adaptive gripper**			
	**Sponge**		**MS-PSA**		**P-HA**		**HA**		**T42**	
	**Grasp**	**Stability**	**Grasp**	**Stability**	**Grasp**	**Stability**	**Grasp**	**Stability**	**Grasp**	**Stability**
Master chef can	N^*^	N^*^	Y	Y	N^*^	N^*^	Y	Y	Y	Y
Soft ball	N^*^	N^*^	Y	Y	N^*^	N^*^	Y	Y	Y	Y
Mustard bottle	Y	Y	Y	Y	Y	Y	Y	Y	Y	Y
Chain	Y	N	Y	Y	Y	Y	Y	Y	Y	Y
Credit card blank	N	N	Y	Y	N	N	N	N	N	N
Fork	N	N	Y	Y	Y	Y	Y	Y	Y	Y
Small cup	Y	Y	Y	Y	Y	Y	Y	Y	Y	Y
Jello box	Y	Y	Y	Y	Y	Y	Y	Y	Y	Y
Wooden cube	Y	Y	Y	Y	Y	Y	Y	Y	Y	Y
Plastic banana	Y	N	Y	Y	Y	Y	Y	Y	Y	N
Racquetball	Y	Y	Y	Y	Y	Y	Y	Y	Y	Y
Marble	Y	N	Y	Y	Y	Y	Y	Y	Y	Y
YCB Score	N/A		392/404		304/404		273/404		356/404	

* *Grasps were not possible, as the object dimensions exceed the gripper aperture*.

**Table 3:** Comparison of different grippers and hands in terms of achievable contact surface area and clench force.

**Gripper**	**Contact surface**		**Max clench force at base**
	**Small cup**	**Mustard**	
Parallel jaw MS-PSA	1,971 mm^2^	2,295 mm^2^	31 N	
Parallel jaw HA	2,754 mm^2^	3,267 mm^2^	30 N	
Parallel jaw Sponge	2,250 mm^2^	4,260 mm^2^	28 N	
HA	3,186 mm^2^	3,645 mm^2^	9 N	
T42	896 mm^2^	3,360 mm^2^	12 N	

**Table 4:** Comparison of the proposed grippers and hands in terms of finger-pad material, type of compliance, weight, link lengths, and number of joints.

**Gripper**	**Finger-pad material**	**Type of compliance**	**Weight**	**Phalanx length**		**# of pin joints**
				**Distal**	**Proximal**	
Parallel jaw MS-PSA	Vytaflex 30	Compliant finger	540 g	180 mm	–	0
Parallel jaw HA	PMC-780	Elastic pin array	625 g	140 mm	–	0
Parallel jaw Sponge	Polyurethane	Sponge pad	468 g	140 mm	–	0
HA	PMC-780	Elastic pin array and finger joints	627 g	65 mm	70 mm	2
T42	Vytaflex 30	Compliant pad and finger joints	503 g	48 mm	63 mm	2
CAM	Vytaflex 30	Compliant pad, finger joints and manipulation mechanisms	478 g	45 mm	65 mm	2

### 5.2. Dexterous, In-hand Manipulation

Preliminary experiments on the CAM fingers demonstrated the potential applications of utilizing structural compliance. Exploiting compliant surfaces for reduced grasping force requirement, the excess motor capacities could be used for other coupled actuation. This design requires redundant motor capacities either by increased motor capabilities or increased passive compliance to reduce required grasp forces on objects. As an extended application of underactuation, we presented the CAM fingers with rotational and linear in hand manipulation capabilities. In Figure 14, manipulation of objects for both in hand grasp and non grasped objects were demonstrated. This design allows dexterous manipulation of objects without external aid from the robot arm. Also, the increased functionality can be easily integrated into existing underactuated designs and does not add extensive weight. The added rotational or translation DOF facilitate the execution of dexterous, in hand manipulation tasks that do not require complex planning. Traditionally, in order to rotate an object similar to operation (b) shown in Figure 14, a robot gripper must grasp the object and re-orient via external arm motion. For a CAM finger, this can be achieved with greater efficiency by applying appropriate tendon forces.

## 6. Conclusion

In this paper, we introduced, analyzed, and experimentally validated a series of alternative uses of structural compliance for the development of simple, adaptive robot hands. Exploratory designs focused on new alternatives to finger pad compliance and joint compliance were presented.

These hands can facilitate and simplify the execution of dexterous tasks (e.g., grasping or dexterous, in-hand manipulation tasks), without requiring sophisticated sensing elements or complicated control laws. More specifically, we proposed pre-shaped, compliant robot fingers that can adapt to different object geometries, extracting robust grasps. Subsequently, we presented a design of hyper-adaptive finger pad that facilitates the maximization of the area of the contact patches between the robot finger and the grasped object, maximizing also the stability of the grasps. These alternative uses of structural compliance designs focusing on increasing grasp stability provided new possibilities from traditional padding approaches. Finally, we introduced the concept of the compliance adjustable manipulation by introducing compliant elements in-series with the robot hand's tendon routing system. The concept extends underactuated mechanisms by appropriately selecting the imposed tendon loads and taking advantage of the adaptive behavior of the system. The efficiency of the proposed concepts and designs was experimentally validated with a variety of experimental paradigms involving the execution of robust grasping and dexterous, in-hand manipulation tasks with both model and everyday life objects. The adaptive behavior of underactuated and compliant robot hands reduces the weight, control complexity, and, consequently, cost of the final device.

In terms of future work, further studies into individual proposed designs are required. Also, validation and analysis of proposed alternative use of structural compliance design should be further investigated. Items such as contact force, minimal contact surface for stable grasp and full YCB benchmark on grippers would be investigated. Finally, we will evaluate adjustable pad applications on proposed alternatives to structural compliance designs in the future.

## Data Availability Statement

All datasets analyzed for this study are included in the article/supplementary material.

## Author Contributions

C-MC designed and manufactured the pre-shaped adaptive robot fingers and the parallel hyper adaptive fingers. LG designed and manufactured the hyper adaptive fingers. AZ and ML worked on the design of the grippers with compliance adjustable manipulation motions. NE was in charge of the analysis on grasp quality measures. C-MC, LG, NE, and ML executed the proposed experiments. ML supervised the project.

### Conflict of Interest

The authors declare that the research was conducted in the absence of any commercial or financial relationships that could be construed as a potential conflict of interest.
